# Cockroaches as Mechanical Vectors of Intestinal Parasites in Sana'a City, Yemen

**DOI:** 10.1155/japr/3583742

**Published:** 2025-09-18

**Authors:** Abbas M. A. Al-Azab, Yousef A. J. Fadladdin, Saeed M. N. Alasmari

**Affiliations:** ^1^Biological Science Department, Faculty of Science, Sana'a University, Yemen; ^2^Department of Biological Sciences, Faculty of Sciences, King Abdulaziz University, Jeddah, Saudi Arabia; ^3^Department of Biology, Faculty of Science and Arts, Najran University, Najran, Saudi Arabia

**Keywords:** cockroaches, intestinal parasites, mechanical, Yemen

## Abstract

This study investigated the role of cockroaches, *Periplaneta americana*, and *Blattella germanica* species as mechanical vector hosts for intestinal parasites in Sana'a City, Yemen, from May to August 2022. Three hundred sixty-five cockroach samples were systematically collected from various sites, including markets, garbage disposal areas, and butcher shops in designated regions. These samples were accurately examined for parasites on their external surfaces. Out of the 365 samples, 198 (54.24%) were infected with a variety of parasites, including protozoa, eggs, and larvae of nematodes. This investigation revealed the presence of nine distinct parasite species on the external surfaces of cockroaches, including three species of protozoan cysts and six species of helminths. Specifically, the identified parasites were *Entamoeba coli* (69, 60%), *Entamoeba histolytica*/*dispar* (61, 58%), *Giardia lamblia* (56, 53%), *Ascaris lumbricoides* (45, 31%), *Hymenolepis nana* (42, 35%), *Enterobius vermicularis* (23, 17%), hookworm (9, 6%), *Trichuris trichiura* (7, 3%), and *Strongyloides stercoralis* (2, 1%) of *P. americana* and *B. germanica*. The dominant parasite among the samples was *E. coli* (69, 60.%), whereas *S. stercoralis* (2, 1%) exhibited the lowest prevalence among the cockroaches collected. Noqum and Assafi'yah zones showed the highest numbers of infected cockroaches, with 71% and 63%, respectively, while the lowest numbers were obtained from Al-Kuwait Hospital (5%). To improve understanding in this area, further investigations would be required to isolate and identify parasites from diverse cockroach specimens' internal (digestive tract) and external surfaces.

## 1. Introduction

Parasitic infections affect both humans and animals worldwide, posing significant health risks and economic burdens [1, 2]. Based on fossil records, cockroaches (Insecta: Blattaria) have persisted on Earth for 305 million years, establishing themselves as the most ubiquitous insect species globally. Renowned for their prevalence, cockroaches are notably prominent as pests within various premises, posing significant concerns regarding food contamination [3–7].

Their widespread presence and adverse impacts on domestic environments consistently position them as a primary pest issue for homeowners. Recent studies indicate the existence of approximately 4500 cockroach species distributed across all regions of the globe [8, 9]. Although primarily concentrated in tropical and subtropical regions, the majority of these species are not classified as pests [10]. Their pervasive presence can be attributed to their remarkable ability to adapt to human environments, leading to an apparent surge in their populations. While 30 species have established associations with human habitats, only a select few tend to live typically in human dwellings [11]. Among the most prevalent cockroach species known to cause issues for humans are *Blattella germanica*, *Periplaneta americana*, and *Blatta orientalis* [12, 13]. Cockroaches frequently scavenge on human waste, refuse, and sewage, facilitating the dissemination of pathogenic agents in their surroundings [3, 14, 15]. They serve as primary mechanical vectors for various pathogenic microorganisms, some of which can lead to severe illnesses in humans and domestic animals. Acknowledged for their pivotal role in the transmission and dissemination of numerous fungi, viruses, protozoa, and fungi and playing a vital role as intermediate hosts for specific pathogenic intestinal worms, they are deemed crucial agents in the spread of significant diseases through both mechanical and biological means [16–18]. Moreover, their nocturnal and unsanitary behaviors render cockroaches optimal carriers of a diverse array of pathogenic microorganisms [14, 19]. Cockroaches not only have the potential to induce food poisoning but also to transmit bacteria, fungi, and protozoa such as cysts of *Giardia lamblia*, *Entamoeba histolytica*, and *Balantidium coli*; oocysts of *Cryptosporidium parvum*, *Cyclospora cayetanensis*, and *Isospora belli*; some worm eggs such as *Ascaris lumbricoides*, *Enterobius vermicularis*, *Trichuris trichiura*, and *Hymenolepis nana* of several pathogenic intestinal worms; and larvae of *Strongyloides stercoralis*, viruses, and other pathogenic microorganisms within contaminated environments [3, 13, 20]. Certain cockroaches exhibit the capability to bite humans, particularly during their sleep [21].

In their study, Pai et al. [22] documented that a proportion of 4% of *P. americana* and 10% of *B. germanica* specimens investigated was found to carry cysts of *E. histolytica/dispar*, situated either on their cuticle or within their digestive tract.

Three predominant cockroach species in Yemen are identified: *B. germanica*, *B. orientalis*, and *P. americana*. Presently, there is a shortage of reliable information and no documented reports regarding the potential role of cockroaches in the transmission of parasites in Sana'a, Yemen. Consequently, this study investigates and identifies external parasites in cockroaches collected from select districts within Sana'a City, Yemen.

There is a lack of documented reports regarding the potential involvement of cockroaches in the transmission of parasites.

## 2. Materials and Methods

### 2.1. Description of Study Site

**Figure 1:**
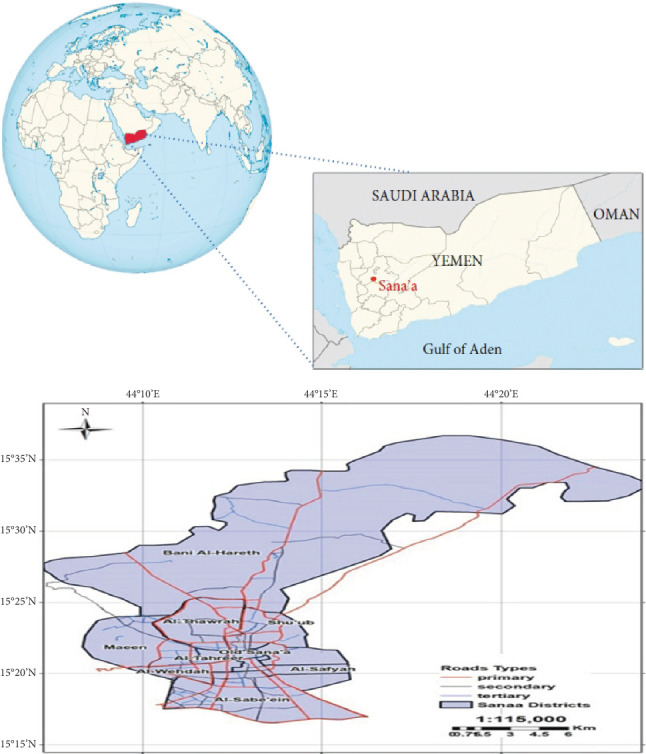
Map of Sana'a City showing study areas and cockroach sampling sites (Yemen Remote Sensing).

This study was carried out in Sana'a, the capital city of Yemen, located at an elevation of 2150 m above sea level, positioned between latitudes 15°70⁣′ and 16°66⁣′ N and longitudes 33°00⁣′ and 48°50⁣′ E. Sana'a experiences cold winters and moderate summers. The study encompassed six specific locations within Sana'a City: Al-Kuwait Hospital, Alqa'a, Hail, Madhbah, Noqum, and Assafi'yah, as illustrated in Figure 1.

### 2.2. Cockroach Sampling and Identification

Over 4 months, a comprehensive total of 365 cockroaches was gathered to identify external parasites. These cockroaches were collected in a convenient way from distinct locations, encompassing indoor and outdoor environments at designated sites in the public markets, open-air markets, using empty jars smeared in a thin layer of Vaseline and lured with pieces of bread soaked in water, sticky traps, and the hand-catching method with sterile gloves [12, 23].

The empty jars and traps were placed strategically in selected sites where cockroaches are likely to be found, such as butcher shops, vegetable markets, and nearby sewage outlets. The selected sites were chosen randomly, and the traps (jars) were not placed haphazardly or without purpose; instead, the traps were positioned with careful planning in specific high-risk spots, reasoning to maximize the chances of catching cockroaches.

Only adult cockroaches with intact bodies were selected for laboratory processing. The collection of cockroaches was conducted every month, spanning from May to August 2022. The specimens were sourced from diverse outlets, including markets and refuse disposal areas. The methodology for collection entailed the utilization of sterile test tubes and sterile gloves, according to [14], between 8.00 a.m. and 2.00 p.m., and strategically positioned traps in specific locations during the evening, which were then retrieved the subsequent morning [23]. Following collection, the cockroach specimens were meticulously transferred into labeled tubes and conveyed to the Medical Entomology Laboratory, Biological Sciences Department, Faculty of Science at Sana'a University. To maintain sterility, the cockroaches were humanely euthanized within a sterile jar utilizing chloroform-soaked cotton. After euthanasia, the cockroaches underwent examination under a dissecting microscope and were identified employing conventional taxonomic keys.

### 2.3. Isolation of Parasites From the External Body of Cockroaches

A standard vial was utilized to isolate the parasites from each cockroach. Two milliliters of normal saline were introduced into the vial, and the solution was vigorously agitated by hand for 2 min. This method facilitated the detachment of parasites adhering to the cockroach's exterior. Subsequently, 1 mm of the rinsing solution was transferred to a centrifuge tube. The tube was then subjected to centrifugation at 2000 rpm for 5 min. The resultant supernatant was discarded while the residual sediment was stained with a 1% Lugol's iodine solution. Following staining, the sediment was examined under a light microscope, utilizing a 40x objective lens, following the protocol outlined by [19, 24, 25]. For the identification of parasites, taxonomic references delineated by [26–28] were employed. These references facilitated the precise classification of the distinct parasite species encountered during the investigative process.

### 2.4. Data Analysis

After collecting the data in this study, the gathered information was entered into Microsoft Excel for subsequent analysis. The analytical process investigated the various intestinal parasites identified from the cockroach specimens. Descriptive analytical methods were utilized to ascertain the frequencies of occurrence and compute the corresponding percentages or prevalence rates of these parasites.

The collected data were statistically analyzed by using variance (ANOVA), with the least significant difference (LSD) test employed for mean comparisons at a significance level of *p* ≤ 0.05 using the SAS software program, Version 9.3 (SAS Institute, 2006). PROGRAM was utilized for these analyses.

## 3. Results

**Figure 2:**
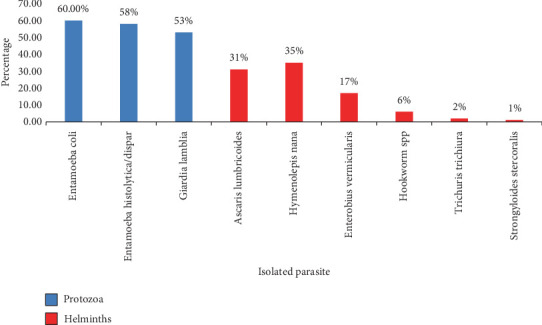
Percentage of parasite species isolated from *B. germanica* in Sana'a City, Yemen, 2022.

**Figure 3:**
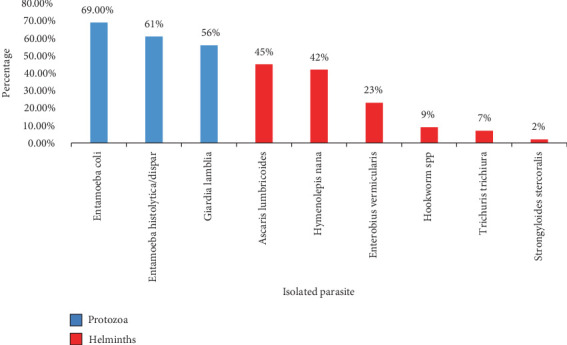
The percentage of parasite species isolated from *Periplaneta americana* in Sana'a City, Yemen, 2022.

Three hundred sixty-five cockroaches were collected and examined for intestinal parasites from six districts in Sana'a City, which is located in Southwestern Yemen. The species *P. americana* and *B. germanica* were collected in this study. As shown in Tables 1, 2, 3, and 4 and Figures 1 and 2 out of the examined samples, 198 cockroaches (54.24%) tested positive for at least one intestinal parasite. Among these, 86 cases (50%) were linked to *B. germanica*, with 148 instances (65.2%) attributed to protozoa, and 79 cases (34.8%) associated with worm eggs and larvae. Conversely, 112 cases (58%) involving *P. americana* exhibited infections, with 208 cases (59.3%) attributed to protozoa and 143 cases (40.7%) to worm eggs and larvae. Notably, nine species of medically and veterinary significant parasites were identified (Figures 2 and 3). Protozoan infections were predominant, while lower incidences were associated with worm eggs and larvae. On the other hand, among the collected cockroaches, the predominant protozoan parasite observed was *Entamoeba coli* (69, 60%) of the infections, following *E. histolytica*/dispar (61, 58%) of the observed infections. Conversely, the lowest prevalence of helminth parasites was observed in *S. stercoralis*, with infection rates of 1.2% in both cockroaches, *P. americana* and *B. germanica*, respectively. In terms of the locations where the infected cockroaches were collected, the highest number of infected specimens was found in Noqum (67%–71%), while the lowest prevalence of parasites was in Al-Kuwait Hospital, with 6%–25% of the collected cockroaches being infected by both cockroaches, *P. americana* and *B. germanica*, respectively.

As shown in Tables 1, 2, 3, and 4, the results indicate that *P. americana* cockroaches have a higher potential as a vector of parasites compared to *B. germanica*. These results are in agreement with prior investigations that have reported similar observations [2, 25, 29].

## 4. Discussion

Cockroaches are considered entomophobic and nuisance insects and play a role as mechanical vectors to transmit various pathogens such as parasites to humans and animals, causing human and zoonotic diseases [29–32].

The results of this study clearly showed that the two species of cockroaches (*P. americana* and *B. germanica*) that were collected from selected sites in the western part of Sana'a, Yemen, carry on their external surfaces human intestinal parasites such as *E. coli* and *E. histolytica*, indicating that concerns about the potential role of these two species of cockroaches as mechanical vectors of pathogens among households living in those areas should not be dismissed.

Our study agreed with several studies conducted around the world on cockroaches [29, 33–36] that demonstrated that cockroaches transmit parasites and their developmental stages.

The results of the current study also agreed with the results of studies conducted in different countries by [23, 37–40], who reported that cockroaches have the potential to become mechanical vehicles for the spread of various zoonotic enteric parasites due to cockroaches' feeding habits and preferences for human food and feces.

In contrast, some previous studies have shown that no major infections transmitted by cockroaches have been recorded [19]. The presence of *E. coli*, *E. histolytica*, and *G. lamblia* may be due to the ability of cysts to transmit infection. These cysts can resist environmental conditions and survive for several weeks outside the host body so that they can reach the cockroaches [23, 41].

The differences in prevalence rates of parasites that are present on cockroaches obtained in this study and previous studies may be attributed to the collection methods, sampling sites, differences in environmental conditions of the study areas, socioeconomic conditions, and health status of the population.

In this study, the higher rate of parasites detected on cockroaches in the areas of Noqum (71%) and Assafi'yah (63%) compared to other sites, such as Al-Kuwait Hospital (6%), Hail, Alqa'a, and Madhbah, may be attributed to the samples collected from crowded markets located in unsanitary conditions and to unsanitary methods of solid waste disposal nearby, which may encourage the spread of cockroaches [42].

The results of this study are supported by the findings of Auta et al. [17], who reported that 95.33% of *P. americana* cockroaches were infected with several species of parasites, as they isolated and identified gastrointestinal parasites, including *S. stercoralis* (25.26%), *E. vermicularis* (13.68%), and *E. histolytica* (12.28%), and mentioned that *P. americana* represents an important reservoir of parasites that can cause disease in humans, as well as cockroaches being potential mechanical transmitters of human nematode and protozoan parasites that may pose threats to public health if not adequately managed. Another study conducted by Tatang et al. [29] agrees with our results, as they reported an overall transport rate of 47.39% from three species of cockroaches: *P. americana*, *B. germanica*, and *B. orientalis*. They identified six parasites, including *Ascaris* (33.76%) and *Trichuris* (11.97%), and mentioned that the parasites were found more commonly on the external surface (54.27%) of cockroaches than in the internal surface (GIT, 38.51%). Al-Aredhi [43] agrees with our study somewhat, as he indicated that eight species of parasites were isolated from the external surface (62%) and the digestive tract (2%) of German cockroaches (*B. germanica*), with an infection rate of 78%. His results showed the presence of three species of parasites shared between humans and animals, carried by the German cockroach: *E. coli cysts*, *E. vermicularis* eggs, and *A. lumbricoides* eggs, which were isolated from the external body of the German cockroach. The infection rate by protozoa was 62%, while the infection rate by worms was 70%. Otu-Bassey et al. [44] revealed that 27.5% tested positive for parasites overall, with more protozoa (22.5%) than helminths (5.0%) (*p* < 0.05). These findings support our findings that the infection rate with protozoa is higher than that of helminths. The parasites detected in their study were *I. belli* (50.8%), *E. vermicularis* (43.1%), *A. lumbricoides* (3.1%), and *E. histolytica* (3.1%).

On the other hand, the lowest number of parasites was isolated on cockroaches collected from Kuwait Hospital, which confirms that effective waste management systems and disinfectants used in hospitals played a role in killing and displacing vectors, thus eliminating food sources, disinfecting the environment such as using rituals that prevent cockroaches from picking up and spreading parasites. Some hospital phenol-based disinfectants act as repellents under certain concentrations, leading to a reduction in the number of cockroaches and thus reducing the number of parasites associated with them.

Finally, *P. americana* cockroaches act as mechanical vectors of parasites when compared with *B. germanica*. Because *P. americana* cockroaches feed on feces and dirt, these cockroaches can spread infections through the fecal–oral route [23, 45]. Furthermore, *E. histolytica* and *G. lamblia* cause diarrhea resulting from amoebiasis and giardiasis. These diseases are named as fatal diseases, especially in children [46]. Some parasites, such as *S. stercoralis*, can cause complex infections and lead to high mortality rates, primarily due to hyperinfection or widespread infection, especially in immunocompromised individuals [47, 48].

## 5. Conclusion

This study's findings indicate that the cockroaches are infected with nine different parasites, including protozoa and helminths. Additionally, many previous studies have confirmed that cockroaches transmit infections to humans, suggesting that both *P. americana* and *B. germanica* can be significant vectors of gastrointestinal parasites. The *P. americana* cockroaches were clearly distinguished by their infection with parasites at the sites selected in this study.

The presence of cockroaches, especially in traditional markets, hospitals, and food-handling areas, should be considered a serious hygiene and infection control concern, as they have significant public health risks due to their ability to pick up and transmit pathogens. Therefore, controlling the cockroach populations and raising awareness will greatly reduce their presence in our environment and eventually lower the transmission of parasites that may lead to various diseases like amoebiasis, giardiasis, ascariasis, hymenolepiasis, enterobiasis, and other gastrointestinal infections, particularly in immunocompromised people and children.

## Figures and Tables

**Table 1:** The percentage of intestinal parasites identified in populations of *B. germanica* in Sana'a City, Yemen, 2022.

**Selected areas**	**No. of cockroaches examined**	**Infected cockroaches,** **n** **(%)**
Al-Kuwait Hospital (the courtyard and the public market)	32	2 (6%)
Hail (public markets)	25	10 (40%)
Alqa'a (public markets)	26	15 (58%)
Madhbah (public markets)	30	20 (67%)
Noqum (public markets)	24	17 (71%)
Assafi'yah (public markets)	35	22 (63%)
Total	172	86 (50%)

**Table 2:** Statistical parameters of isolated populations of *B. germanica* in Sana'a City, Yemen, 2022.

**Parasite/location**	**Statistical param**	**Al-Kuwait Hospital (the courtyard and public markets)**	**Hail zone (public markets)**	**Alqa'a zone (public markets)**	**Madhbah (public markets)**	**Noqum (public markets)**	**Assafi'yah (public markets)**	**p** **value**	**R** ^2^
*Entamoeba coli*	Mean ± SE	1.00 ± 0.33^b^	0.0 ± 0.33^b^	0.0 ± 0.0^c^	1.00 ± 0.33^c^	1.00 ± 0.33^b^	0.0 ± 0.0^b^	0.229	0.402
	95% CI	−0.77 to 2.10	−1.10 to 1.77	0.0–0.0	−0.77 to 2.10	−0.77 to 2.10	0.0–0.0		
*Entamoeba histolytica/dispar*	Mean ± SE	3.00 ± 0.67^a^	3.00 ± 0.0^a^	3.00 ± 0.33^ba^	1.00 ± 0.33^bac^	1.00 ± 0.33^b^	0.0 ± 0.33^b^	0.089	0.511
	95% CI	0.79–6.54	3.00–3.00	1.23–4.10	−0.10 to 2.77	−0.77 to 2.10	−1.10 to 1.77		
*Giardia lamblia*	Mean ± SE	2.00 ± 0.33^ba^	3.00 ± 0.0^a^	2.00 ± 0.33^bc^	1.00 ± 0.00*b*^c^	1.00 ± 0.33^b^	0.0 ± 0.0^b^	0.001	0.779
	95% CI	0.89–3.77	3.00–3.00	0.23–3.10	1.00–1.00	−0.77 to 2.10	0.0–0.0		
*Ascaris lumbricoides*	Mean ± SE	4.00 ± 0.0^a^	3.00 ± 0.33^ba^	3.00 ± 0.88^ba^	2.00 ± 0.58^bac^	2.00 ± 0.33^ab^	0.00 ± 0.67^ab^	0.0825	0.519
	95% CI	4.00–4.00	1.23–4.10	−0.13 to 6.46	−0.48 to 4.48	−0.23 to 3.10	−2.20 to 3.54		
*Hymenolepis nana*	Mean ± SE	2.00 ± 1.33^ba^	4.00 ± 0.58^a^	4.00 ± 067^a^	2.00 ± 0.67^a^	3.00 ± 0.00^a^	2.00 ± 0.88^a^	0.091	0.509
	95% CI	−2.40 to 9.07	1.52–6.48	1.47–7.20	−2.01 to 5.53	1.23–4.10	−1.46 to 6.13		
*Enterobius vermicularis*	Mean ± SE	4.00 ± 1.77^ba^	5.00 ± 186^a^	5.00 ± 0.56^a^	3.00 ± 0.67^ab^	3.00 ± 1.20^ab^	2.00 ± 0.88^ab^	0.079	0.522
	95% CI	−4.26 to 10.92	−4.32 to 11.65	1.52–6.48	−0.5.3 to 5.20	−2.84 to 7.50	−2.13 to 5.46		
Hookworm	Mean ± SE	0.0 ± 0.0^a^	0.0 ± 0.0^a^	0.0 ± 0.0^a^	0.0 ± 0.0^a^	0.0 ± 1.00^a^	0.0 ± 0.67^a^	0.561	0.254
	95% CI	0.0–0.0	0.0–0.0	0.0–0.0	0.0–0.0	−3.30 to 5.30	−2.20 to 3.54		
*Trichuris trichiura*	Mean ± SE	0.0 ± 0.0^a^	0.0 ± 0.33^a^	0.0 ± 0.00^a^	0.0 ± 0.00^a^	0.0 ± 0.33^a^	0.0 ± 0.0^a^	0.571	0.250
	95% CI	0.0–0.0	−1.10 to 1.77	0.0–0.0	0.0–0.0	−0.77 to 2.10	0.0–0.0		
*Strongyloides stercoralis*	Mean ± SE	0.0 ± 0.0^a^	0.0 ± 0.33^a^	0.0 ± 0.00^a^	0.0 ± 0.0^a^	0.0 ± 0.00^a^	0.0 ± 0.0^a^	0.458	0.294
	95% CI	0.0–0.0	−1.10 to 1.77	0.0–0.0	0.0–0.0	0.0–0.0	0.0–0.0		

*Note:* Means with the same letter are not significantly different (*p* > 0.05).

**Table 3:** The percentage of intestinal parasites isolated from populations of *Periplaneta americana* in Sana'a City, Yemen, 2022.

**Selected areas**	**No. of cockroaches examined**	**Infected cockroaches,** **n** **(%)**
Al-Kuwait Hospital	20	5 (25%)
Hail	30	20 (67%)
Alqa'a	25	14 (56%)
Madhbah	40	23 (58%)
Noqum	33	22 (67%)
Assafi'yah	45	28 (62%)
Total	193	112 (58%)

**Table 4:** Statistical parameters of parasite species isolated from *P. americana* in Sana'a City, Yemen, 2022.

**Parasite/location**	**Statistical param**	**Al-Kuwait Hospital**	**Hail zone**	**Alqa'a zone**	**Madhbah**	**Noqum**	**Assafi'yah**	**p** **value**	**R** ^2^
*Entamoeba coli*	Mean ± SE	1.00 ± 00^d^	4.00 ± 1.52^c^	5.0 ± 0.88^cb^	3.0 ± 0.33^c^	7.0 ± 0.33^a^	6.0 ± 0.0^ba^	0.0001	0.885
	95% CI	1.00–1.00	1.52–6.48	0.87–8.46	1.89–4.76	5.23–8.10	6.00–6.00		
*Entamoeba histolytica/dispar*	Mean ± SE	2.0 ± 0.33^d^	4.00 ± 0.58^c^	1.00 ± 0.0^d^	5.00 ± 0.33^b^	5.00 ± 0.33^b^	7.00 ± 0.33^a^	0.0001	0.956
	95% CI	0.23–3.10	1.52–6.48	1.00–1.00	3.23–6.10	3.90–6.77	5.23–8.10		
*Giardia lamblia*	Mean ± SE	0.00 ± 0.33^d^	4.00 ± 0.67^bc^	2.00 ± 0.00^dc^	5.00 ± 0.0^ba^	7.00 ± 1.53^a^	4.00 ± 0.33^ba^	0.0012	0.782
	95% CI	−1.10 to 1.77	0.46–6.20	2.00–2.00^cd^	5.00–5.00	−0.57 to 12.57	2.89–5.77		
*Ascaris lumbricoides*	Mean ± SE	0.00 ± 0.33^d^	3.00 ± 0.67^ba^	2.00 ± 0.33^c^	3.00 ± 0.33^cb^	4.00 ± 1.33^a^	3.00 ± 0.33^ba^	0.0002	0.844
	95% CI	1.10–1.77	0.79–6.54	0.23–3.10	1.23–4.10	−2.40 to 9.07	1.23–4.10		
*Hymenolepis nana*	Mean ± SE	1.00 ± 0.33^b^	3.0 ± 0.0^a^	3.00 ± 0.0^a^	2.00 ± 1.33^a^	3.00 ± 0.00^a^	3.00 ± 0.0^a^	0.059	0.550
	95% CI	−0.77 to 2.10	3.00–3.00	3.00–3.00	−2.40 to 9.07	3.00–3.00	3.00–3.00		
*Enterobius vermicularis*	Mean ± SE	0.0 ± 0.0^c^	1.00 ± 0.33^c^	1.00 ± 0.33^c^	1.00 ± 0.33^bc^	2.00 ± 1.33^a^	3.00 ± 0.33^ba^	0.015	0.653
	95% CI	0.0–0.0	−0.77 to 2.10	−0.77 to 2.10	−0.10 to 2.77	−2.40 to 9.07	1.23–4.10		
Hookworm	Mean ± SE	0.0 ± 0.0^c^	0.0 ± 0.33^bc^	0.0 ± 0.0^c^	1.00 ± 0.33^cab^	1.00 ± 0.33^a^	1.00 ± 0.0^cba^	0.008	0.689
	95% CI	0.0–0.0	−1.10 to 1.77	0.0–0.0	−0.77 to 2.10	−0.10 to 2.77	1.00–1.00		
*Trichuris trichiura*	Mean ± SE	0.0 ± 0.0^a^	0.0 ± 0.33^a^	0.0 ± 0.33^a^	0.0 ± 0.67^a^	1.00 ± 0.33^a^	1.00 ± 0.33^a^	0.771	0.172
	95% CI	0.0–0.0	−1.10 to 1.77	−1.10 to 1.77	−2.20 to 3.53	−0.77 to 2.10	−0.77 to 2.10		
*Strongyloides stercoralis*	Mean ± SE	0.0 ± 0.0^a^	0.0 ± 0.0^a^	0.0 ± 0.33^a^	0.0 ± 0.0^a^	0.0 ± 0.33^a^	0.0 ± 0.0^a^	0.571	0.250
	95% CI	0.0–0.0	0.0–0.0	−1.10 to 1.77	0.0–0.0	−1.10 to 1.77	0.0–0.0		

*Note:* There is no significant difference (*p* > 0.05) among means sharing the same letter.

## Data Availability

The data that support the findings of this study are available from the corresponding author upon reasonable request.

## References

[1] Pullan R. L., Smith J. L., Jasrasaria R., Brooker S. J. (2014). Global Numbers of Infection and Disease Burden of Soil Transmitted Helminth Infections in 2010. *Parasites & Vectors*.

[2] Ohanu C. M. G., Nwangwu C. P., Eze S. C., Ekeh F. N. (2024). Parasites of Cockroaches in the University of Nigeria, Nsukka, Enugu State, Nigeria. *Animal Research International*.

[3] Al-bayati N. Y., Al-Ubaidi A. S., Al-Ubaidi I. K. (2011). Risks Associated With Cockroach Periplaneta americana as a Transmitter of Pathogen Agents. *Diyala Journal of Medicine*.

[4] Atiokeng Tatang R. J., Tsila H. G., Wabo Poné J. (2017). Medically Important Parasites Carried by Cockroaches in Melong Subdivision, Littoral, Cameroon. *Journal of Parasitology Research*.

[5] Donkor E. S. (2020). Cockroaches and Food-Borne Pathogens. *Environmental Health Insights*.

[6] Maji A., Ahmed U. A. (2023). Identification of Parasites of Public Health Important From the Body of Cockroach (*Periplaneta Americana*) in Kafin Hausa Area of Jigawa State. *African Journal of Advances in Science and Technology Research*.

[7] Liu J., Yuan Y., Feng L. (2024). Intestinal Pathogens Detected in Cockroach Species Within Different Food-Related Environment in Pudong, China. *Scientific Reports*.

[8] Gondhalekar A. D., Appel A. G., Thomas G. M., Romero A. (2021). A Review of Alternative Management Tactics Employed for the Control of Various Cockroach Species (Order: Blattodea) in the USA. *Insects*.

[9] Adedara I. A., Mohammed K. A., da-Silva O. F. (2022). Utility of Cockroach as a Model Organism in the Assessment of Toxicological Impacts of Environmental Pollutants. *Environmental Advances*.

[10] Lee C. Y., Lee L. C. (2000). Diversity of Cockroach Species and Effect of Sanitation on Level of Cockroach Infestation in Residential Premises. *Tropical Biomedicine*.

[11] Hamu H., Debalke S., Zemene E., Birlie B., Mekonnen Z., Yewhalaw D. (2014). Isolation of Intestinal Parasites of Public Health Importance From Cockroaches *(Blattella germanica*) in Jimma Town, Southwestern Ethiopia. *Journal of parasitology Research*.

[12] Fazeli-Dinan M., Habibi A., Haghi S. F. M., Nikookar S. H., Yazdani-Charati J., Enayati A. (2022). Determination of Susceptibility Levels of Three Different Cockroach Species Including Hospitals German Cockroach, *Blattella germanica* L. (Blattodea: Blattellidae), to Common Insecticides, Cypermethrin, Propoxur and Fenitrothion. *International Journal of Health Sciences*.

[13] Vazirianzadeh B., Mehdinejad M., Dehghani R. (2009). Identification of Bacteria Which Possible Transmitted by *Polyphaga aegyptica* (Blattodea: Blattidae) in the Region of Ahvaz, SW Iran. *Jundishapur Journal of Microbiology*.

[14] Sosan M. B., Ajibade R. O., Adeleye A. O. (2019). Survey of the Distribution and Diversity of Cockroaches (Insecta: Blattaria) on the Campus of a Higher Institution in South-Western Nigeria. *International Journal of Applied Biological Research*.

[15] Fotouhi-Ardakani R., Kababian M., Saghafipour A., Alirezaei M., Vatandoost H. (2022). Molecular Evaluation of the Novel Coronavirus Infection of Cockroaches and Flies Collected From Kamkar-Arabnia Hospital in Qom City, Central Iran: With Innovated Internal Control. *Journal of Arthropod-Borne Diseases*.

[16] Yusof A. M. (2018). Identification of Cockroaches as Mechanical Vector for Parasitic Infections and Infestations in Kuantan, Malaysia. *Journal of Entomology*.

[17] Auta T., Yantaba H. S., Everest A. (2019). Determination of Parasitic Agents Associated With Cockroaches in Dutsin-Ma Town, Northwestern Nigeria. *South Asian Journal of Parasitology*.

[18] Van Woerden H. C., Martínez-Girón R., Martínez-Torre C. (2020). Protozoan Cysts in Faecal Pellets of German Cockroaches (*Blattella germanica*), With Particular Emphasis on *Lophomonas blattarum*. *Acta Parasitologica*.

[19] Salehzadeh A., Tavacol P., Mahjub H. (2007). Bacterial, Fungal and Parasitic Contamination of Cockroaches in Public Hospitals of Hamadan, Iran. *Journal of Vector Borne Diseases*.

[20] Yahaya Z. S., Izzaudin N. A. I., Razak A. F. A. (2017). Parasitic Gregarine blattarum Found Infecting American Cockroaches, *Periplaneta americana*, in a Population in Pulau Pinang, Malaysia. *Tropical Life Sciences Research*.

[21] Okafor-Elenwo E. J., Elenwo A. C. (2014). Human Infecting Parasitic Worms, in Cockroaches From Odau in the Niger Delta Region of Nigeria. *International Journal of Natural Sciences Research*.

[22] Pai H. H., Ko Y. C., Chen E. R. (2003). Cockroaches (*Periplaneta americana* and *Blattella germanica*) as Potential Mechanical Disseminators of *Entamoeba histolytica*. *Acta Tropica*.

[23] Adenusi A. A., Akinyemi M. I., Akinsanya D. (2018). Domiciliary Cockroaches as Carriers of Human Intestinal Parasites in Lagos Metropolis, Southwest Nigeria: Implications for Public Health. *Journal of Arthropod-Borne Diseases*.

[24] Ejimadu L. C., Goselle O. N., Ahmadu Y. M., James-Rugu N. N. (2015). Specialization of *Periplaneta americana* (American Cockroach) and Blattella germanica (German Cockroach) Towards Intestinal Parasites: A Public Health Concern. *IOSR Journal of Pharmacy and Biological Sciences (IOSR-JPBS)*.

[25] Dokmaikaw A., Suntaravitun P. (2020). Prevalence of Parasitic Contamination of Cockroaches Collected From Fresh Markets in Chachoengsao Province, Thailand. *Kobe Journal of Medical Sciences*.

[26] Cheesbrough M. (2010). *District Laboratory Practice in Tropical Countries, Second edition, Part I*.

[27] Lee J. J., Leedale G. F., Bradbury P. (2000). *An Illustrated Guide to the Protozoa, second edition*.

[28] Montresor A. (2019). *Bench Aids for the Diagnosis of Intestinal Parasites*.

[29] Atiokeng Tatang R. J., Tsila H. G., Wabo Poné J. (2017). Medically Important Parasites Carried by Cockroaches in Melong Subdivision, Littoral, Cameroon. *Journal of Parasitology Research*.

[30] Siagian F. E., Livina J., Dana I. M. B. S., Daroedono E., Ronny R. (2017). Prevalence of Cockroaches in A Private Faculty Building/its Surrounding, with Emphasize on its Vectorial Capacity For Intestinal Parasite, Its Public Health Implication and Comparison of the Performance of several Traditional Baits. *Journal of Medical Science*.

[31] Haile T., Mariam A. T., Kiros S., Teffera Z. (2018). Cockroaches as Carriers of Human Gastrointestinal Parasites in Wolkite Town, Southwestern Ethiopia. *Journal of Parasitology and Vector Biology*.

[32] Shaibu M. A., Idris M., Otori M. O., Anchau Z. G. (2019). Detection of Parasites From the External Body Surface of Cockroaches in Female Hostels of Ahmadu Bello University Zaria. *Bayero Journal of Pure and Applied Sciences*.

[33] Ozawa S., Hasegawa K. (2018). Broad Infectivity of *Leidynema appendiculatum* (Nematoda: Oxyurida: Thelastomatidae) Parasite of the Smokybrown Cockroach *Periplaneta fuliginosa* (Blattodea: Blattidae). *Ecology and Evolution Journal*.

[34] Abu-Zaid S. A. S. (2018). Study of the Types of Parasites Transmitted by German Cockroaches Mechanically on Their External Surfaces and Inside Their Intestines. *Journal of Applied Sciences*.

[35] Ziane M., Bouamra M., Berroukeche A. (2022). Biodiversity of Intestinal Parasites Carried by the External Body of Cockroaches at Different Food Locations : Case of Ain TéMouchent City. *Genetics & Biodiversity Journal*.

[36] Al-Badrani S., Al-Rubaye F., Abbass L. (2024). Isolation and Diagnosis of Parasites From Domestic Cockroach *Periplaneta americana* in Mosul City, Iraq. *Journal of Life and Bio Sciences Research*.

[37] Edwin I. (2019). Spatial Distribution and Prevalence of Parasites Vectored by *Periplaneta americana* in Southern, Nigeria: Implication for Intervention. *Asian Journal of Biological Sciences*.

[38] Bala A. Y., Sule H. (2012). Vectorial Potential of Cockroaches in Transmitting Parasites of Medical Importance in Arkilla, Sokoto, Nigeria. *Nigerian Journal of Basic and Applied Sciences*.

[39] Nedelchev S., Pilarska D., Takov D., Golemansky V. (2013). Protozoan and Nematode Parasites of the American Coakroach *Periplaneta americana* (L.) From Bulgaria. *Acta Zoologica Bulgarica Journal*.

[40] Hayati R. Z., Susanna D. (2020). The Human Pathogens Carried by the Cockroaches in the Food-Related Environment Potentially Causing a Foodborne Diseases: A Systematic Review. *Malaysian Journal of Public Health Medicine*.

[41] Oyeyemi O., Agbaje M., Okelue U. (2016). Food-Borne Human Parasitic Pathogens Associated With Household Cockroaches and houseflies in Nigeria. *Parasite Epidemiology and Control*.

[42] Ngwawe C. (2017). Assessment of Cockroach Infestation Levels, Awareness and Control Practices of Vendors in Ready-to-Eat Food Premises in Kisumu City, Kisumu County. *American Journal of Humanities and Social Sciences Research*.

[43] Al-Aredhi H. S. (2014). Isolation and Identification of Parasites Transmitted by Cockroach *Blattella germanica* in Al-Diwaniya City/lraq. *Muthanna Journal of Pure Science*.

[44] Otu-Bassey I. B., Mbah M., Udoh D. I., Kayode J. O. (2019). Parasitological Survey of Domestic Cockroaches in Some Residential Houses in Calabar, Nigeria. *Calabar Journal of Health Sciences*.

[45] Guzman J., Vilcinskas A. (2020). Bacteria Associated With Cockroaches: Health Risk or Biotechnological Opportunity?. *Applied Microbiology and Biotechnology*.

[46] Dhubyan Mohammed Zaki Z. (2022). Prevalence of Entamoeba histolytica and *Giardia lamblia* Associated With Diarrhea in Children Referring to lbn Al-Atheer Hospital in Mosul, Iraq. *Archives of Razi institute*.

[47] Vasquez-Rios G., Pineda-Reyes R., Pineda-Reyes J., Marin R., Ruiz E. F., Terashima A. (2019). *Strongyloides stercoralis* Hyperinfection Syndrome: A Deeper Understanding of a Neglected Disease. *Journal of Parasitic Diseases*.

[48] Yeh M. Y., Aggarwal S., Carrig M. (2023). *Strongyloides stercoralis* Infection in Humans: A Narrative Review of the Most Neglected Parasitic Disease. *Cureus*.

